# Case of Stroke from Cerebral Vasculitis following Carfilzomib, Lenalidomide, and Dexamethasone Therapy in a Patient with Relapsing Multiple Myeloma

**DOI:** 10.1155/2019/5180424

**Published:** 2019-11-23

**Authors:** Deborah Osafehinti, Kaveh Zivari

**Affiliations:** Department of Internal Medicine, Maimonides Medical Center, Brooklyn, NY, USA

## Abstract

Lenalidomide, a synthetic derivation of thalidomide, in recent years, has been the backbone of multiple myeloma treatment leading to improved survival. Common adverse effects from lenalidomide-based regimens include hypertension, heart disease, and venous thromboembolism. Hence, thromboprophylaxis is recommended to reduce the risk of stroke. We report a case of stroke from cerebral vasculitis in a patient receiving carfilzomib, lenalidomide, and dexamethasone for relapsing multiple myeloma, not previously published. Medical oncologists should be aware of other causes of stroke among multiple myeloma patients receiving a lenalidomide-based regimen to prevent its occurrence.

## 1. Introduction

Multiple myeloma (MM) is a hematological malignancy characterized by the neoplastic proliferation of plasma cells leading to monoclonal immunoglobulin production [1–3]. It accounts for 10 to 15% of all hematological malignancies [2, 4], and in the United States, there are more than 20,000 new cases of multiple myeloma every year [4]. Treatment choice is mainly dependent on the functional status of the patient and the aggressiveness of the disease. These include autologous stem cell transplant (for those who are transplant eligible) and chemotherapy [3, 4]. The overall survival rates and treatment response have improved over the past decade following the introduction of novel targeted therapies such as the immunomodulatory agents (IMiD), thalidomide, and proteasome inhibitors (PI), bortezomib [5, 6]. However, MM remains an incurable disease, and many patients will relapse despite receiving appropriate treatment.

The US FDA recently approved the combination of carfilzomib (Kyprolis), a proteasome inhibitor, lenalidomide (Revlimid), a synthetic derivative of thalidomide [7], and dexamethasone for patients with relapsed/refractory multiple myeloma after reports showed better survival outcomes compared to earlier regimens [8, 9]. In the ASPIRE trial, patients treated with the carfilzomib (Kyprolis), lenalidomide (Revlimid), and dexamethasone regimen (i.e., KRd regimen) resulted in a progression-free survival median of 26.3 months compared to 17.6 months in patients treated with lenalidomide and dexamethasone (Rd) regimen [9]. However, grade≥3 adverse effects and serious effects were higher in the KRd arm than the Rd arm, some of which include hypertension, ischemic heart disease, heart failure, and venous thromboembolism [9, 10]. Specifically, by substituting an amino group in the phthaloyl ring, lenalidomide has less adverse side effects compared to thalidomide [11]. The same change in the phthaloyl ring of lenalidomide results in a complex immune modulation effect that is responsible for its activity. However, this complex can lead to lenalidomide toxicity. Most cases of lenalidomide toxicity include skin lesions [11–14], urticaria [15], but rarely vasculitis [13]. We report a case of a patient on the KRd regimen who presented with stroke caused by cerebral vasculitis.

## 2. Case

We present a 59-year-old female with a history of multiple myeloma diagnosis in 2015. Laboratory results at the time of diagnosis had shown hemoglobin of 6.4 g/dl, platelet of 43,000/Ul, and creatinine of 1.2 mg/dl increased from her baseline of 0.7 mg/dl. Serum protein electrophoresis showed an immunoglobulin A level of 9680 mg/dl with low immunoglobulin G and M levels. She had induction chemotherapy with cyclophosphamide, bortezomib, and dexamethasone (CyBorD) regimen, received autologous bone marrow transplantation in 2016, and subsequently went into remission. Unfortunately, she relapsed in 2017 and then started a salvage regimen with carfilzomib, lenalidomide, and dexamethasone every two weeks to 1 month as well as daily low-dose aspirin.

**Figure 1:**
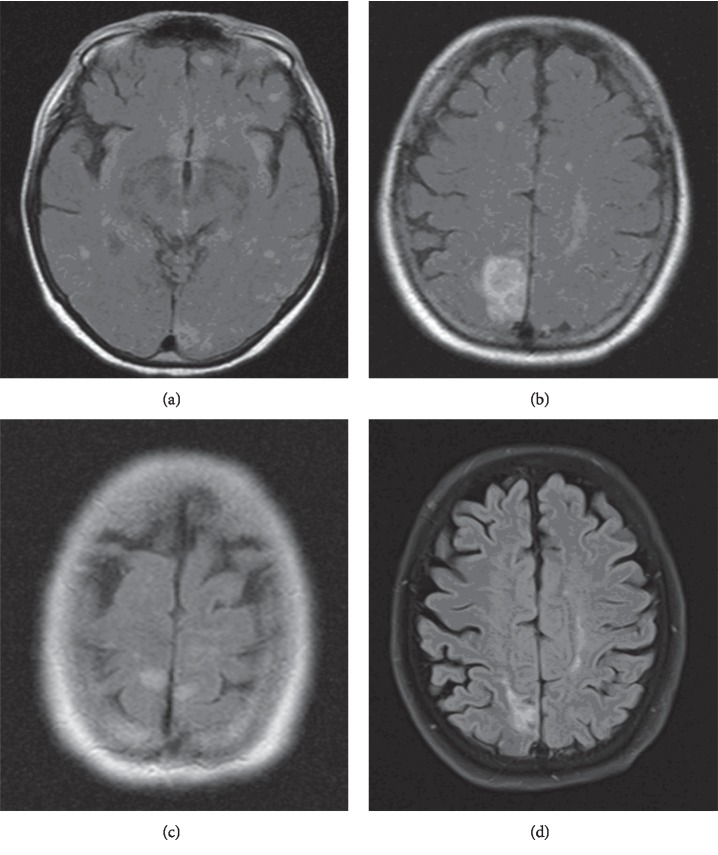
MRI brain showing acute ischemic regions. (a-b) Acute infarcts in the posterior circulation involving bilateral cerebellar hemispheres, medial bilateral occipital and parietal lobes, and the posterior watershed region at the vertex with small amount of hemorrhagic conversion within the right parietal parasagittal lesion. (c-d) MRI image of infarct in the medial occipital lobe and parietal lobes bilaterally.

**Figure 2:**
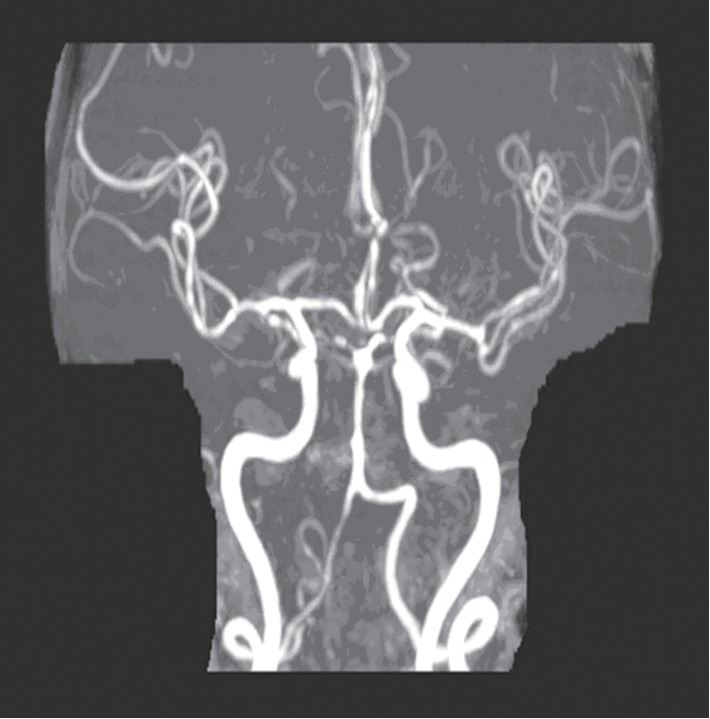
MRA showing multiple luminal narrowing beaded pattern in most vessels (anterior cerebral arteries, posterior inferior M2 division of the right middle cerebral artery, and posterior cerebral arteries).

In February 2019, she was admitted to our hospital with complaints of increased lethargy, multiple falls, and worsening headaches for 4 to 5 days. Physical examination showed mild left facial droop. Aside from being a current everyday smoker (about five cigarette sticks per day) with COPD and a former heroin abuser on a methadone maintenance program, she had no history of diabetes, hypertension, or previous strokes. CT of the head revealed a mass-like structure in the right precentral region measuring approximately 1.5 cm by 0.9 cm surrounded by a large area of vasogenic edema without midline shift and a small age-indeterminate infarct in the left posterior paramedian parietal lobe. MRI of the brain (Figure 1) revealed nonenhancing acute infarcts in the posterior circulation involving bilateral cerebellar hemispheres, medial bilateral occipital and parietal lobes, and the posterior watershed region at the vertex with small amount of hemorrhagic conversion within the right parietal parasagittal lesion. MRA of the brain (Figure 2) without contrast further revealed multiple long and short segments of luminal narrowing with a beaded pattern in the anterior cerebral arteries, posterior inferior M2 division of the right middle cerebral artery, and posterior cerebral arteries indicating vasculitis as the etiology. CRP was more than 40 mg/dl. dsDNA and ANCA testing were both negative. A thromboembolic phenomenon was ruled-out as transthoracic echocardiography showed no evidence of valvular disease, vegetation, patent foramen ovale, or thrombosis. Also, carotid Doppler showed no significant carotid stenosis.

We withheld further chemotherapy, and a short course of dexamethasone IV was started and later switched to prednisone PO before discharge. She also received anticoagulation with apixaban. After outpatient follow-up, she resumed carfilzomib, cyclophosphamide, and dexamethasone regimen, which responded briefly as M spike returned to near baseline but later developed myelomatous meningitis. As a result, she received intrathecal methotrexate and cytarabine and commenced salvage therapy with DCEP (dexamethasone, cyclophosphamide, etoposide, and cisplatin). Three weeks after receiving the first cycle, she was admitted for severe pancytopenia, septic shock with *Klebsiella* bacteremia, acute respiratory distress syndrome (ARDS), and gastrointestinal bleeding secondary to thrombocytopenia and apixaban use. Unfortunately, she died from multiorgan failure.

## 3. Discussion

There have been no prior reports of stroke in a patient with relapsed multiple myeloma treated with carfilzomib, lenalidomide, and dexamethasone regimen [8, 9]. However, the lenalidomide and dexamethasone regimen approved for patients with newly diagnosed multiple myeloma has an increased risk for arterial thromboembolism, including stroke and myocardial infarction [16, 17]. Lenalidomide carries a black box warning for thromboembolic risks, and thromboprophylaxis with either low-molecular-weight heparin, warfarin, or low-dose aspirin is recommended for patients treated with a lenalidomide-based regimen [18–20]. Our patient, however, had a stroke with cerebral vasculitis being the underlying etiology.

Although the KRd regimen may have a promising safety profile from previous clinical trials, we expect that market surveillance will offer more data about rare or idiosyncratic adverse effects. We hope that such knowledge will identify patients who are at high risk and provide preventive measures to improve their quality of life.
